# Gender and Socioeconomic Disparities in Global Burden of Epilepsy: An Analysis of Time Trends From 1990 to 2017

**DOI:** 10.3389/fneur.2021.643450

**Published:** 2021-04-16

**Authors:** Yin Hu, Yi Shan, Qiang Du, Yao Ding, Chunhong Shen, Shuang Wang, Meiping Ding, Yufeng Xu

**Affiliations:** ^1^Department of Neurology, The Second Affiliated Hospital, College of Medicine, Zhejiang University, Hangzhou, China; ^2^Department of Ophthalmology, The Second Affiliated Hospital, College of Medicine, Zhejiang University, Hangzhou, China

**Keywords:** global burden of disease, epilepsy, time trend, gender disparity, socioeconomic disparity

## Abstract

**Objective:** The objective of the study is to investigate the gender and socioeconomic disparities in the global burden of epilepsy by prevalence and disability-adjusted life-years (DALYs).

**Methods:** The global, regional, and national gender-specific prevalence and DALYs caused by epilepsy by year and age were extracted from the Global Burden of Disease (GBD) Study 2017. The Gini coefficient and concentration index (CI) were calculated to demonstrate the trends in between-country inequality in the epilepsy burden from 1990 to 2017. Paired Wilcoxon signed rank test, Pearson correlation, and linear regression analyses were performed to analyze the association of gender disparity in epilepsy and socio-demographic index (SDI).

**Results:** The DALYs number of epilepsies increased from 1990 to 2017 by 13.8%, whereas age-standardized DALY rates showed a substantial reduction (16.1%). Men had a higher epilepsy burden than women of the same period. The epilepsy burden appeared to be higher in countries with lower socioeconomic development (CI < 0). The Gini coefficient decreased from 0.273 in 1995 to 0.259 in 2017, representing a decline in the between-country gap. Age-standardized prevalence and DALY rates of men were higher than those of women in each SDI-based country group (*p* < 0.0001). Male-minus-female difference (*r* = −0.5100, *p* < 0.0001) and male-to-female ratio (*r* = −0.3087, *p* < 0.0001) of age-standardized DALY rates were negatively correlated with SDI.

**Conclusion:** Although global health care of epilepsy is in progress, the epilepsy burden was concentrated in males and developing countries. Our findings highlight the importance of formulating gender-sensitive health policies and providing more services in developing countries.

## Introduction

Epilepsy is one of the most common and serious neurological diseases, which remains an important cause of disability and mortality, affecting 50 million people worldwide (1). It is defined as a brain disorder characterized by an enduring predisposition to generate epileptic seizure (2). Epilepsy affects people of all ages, particularly prevalent among infants and older age groups (1). Health care (long-term treatment, hospitalization, and surgery) and social services (social support and health education) lead to high health costs (3). Therefore, in many parts of the world, individuals living with epilepsy and their families suffer from a high economic burden for health systems (4). Besides, the lives of patients with epilepsy are impacted with social stigma and discrimination (5).

Epilepsy burden has been measured by calculating disability-adjusted life-years (DALYs; a summary measure of health loss defined by the sum of years of life lost due to premature mortality and years lived with disability) in the Global Burden of Disease (GBD) Study (6–8). Several studies have analyzed the gender difference and socioeconomic disparity in epilepsy. In the GBD 2015 study (9), epilepsy contributed to 5.0% of total DALYs due to neurological disorders and 1.3% of all deaths, with higher DALY and prevalence rates in males. Worldwide, men had a higher incidence of epilepsy compared with women (10). Men were likely to be vulnerable to common risk factors such as brain damage (11). In addition, women tended to have a lower clinical consultation rate than men, especially in counties with lower socioeconomic status (10, 12). Socioeconomic status is related to inequality in the quality of medical care. Leonardi et al. (13) first attempted to quantify the global disparity in the burden of epilepsy based on the GBD 2000 study. More than 80% of people with epilepsy lived in developing countries where epilepsy was not well-treated (14). The previous studies have demonstrated that the lower socioeconomic status group were more likely to develop epilepsy (15, 16). The incidence of epilepsy in low-/middle-income countries (LMICs) was 139.0 (95% confidence interval 69.4–278.2), while it was 48.9 (95% confidence interval 39.0–61.1) for high-income countries (HICs) (17, 18). This association might be a result of less expenditure on health care, lower education level, as well as a higher incidence of risk factors such as infections and traumatic brain injury (19, 20).

Gender and socioeconomic disparities in epilepsy burden are worth more attention in reducing the progression of epilepsy. Existing studies have focused on the relationship between gender or socioeconomic development and the prevalence and incidence of epilepsy. However, there are few studies quantifying gender and socioeconomic disparities in global epilepsy burden in multiple dimensions. Epilepsy affects people of all ages, sexes, races, income groups, and geographical locations. About half of the people with epilepsy have physical or mental illnesses. Physical and mental comorbidities in people with epilepsy are related to poorer health, increased health care demand, decreased quality of life, and greater social exclusion (9). Our study analyzed the global burden of epilepsy by year, age, sex, geography, and socioeconomic status using prevalence and DALYs. In this study, we aimed to assess the gender differences and compare the epilepsy burden across countries with different socioeconomic status, using the most recent data from the GBD 2017 study.

## Methods

### Data Source

The GBD category of epilepsy is defined by the *International Classification of Diseases (ICD)-10* code G40 and G41. The GBD 2017 study collected data from typical surveys from 195 countries and territories based on 354 diseases and injuries from 1990 to 2017. The GBD collaboration quantified health loss by age, sex, and geography over time, using estimated prevalence and DALYs. Methods to calculate DALYs estimates for the GBD 2017 study have been reported previously (21–24). We extracted data from the Global Health Data Exchange (http://ghdx.healthdata.org/gbd-data-tool) based on the GBD 2017 study, including (1) global total and gender-specific burden due to epilepsy, containing prevalence and DALYs number, prevalence and DALYs per 100,000 population (crude rate) and age-standardized prevalence and DALYs rate from 1990 to 2017; (2) global total and gender-specific prevalence and DALYs rate by age group in 2017; (3) gender-specific age-standardized prevalence and DALYs rate in 21 GBD regions in 1990 and 2017; (4) DALYs number and age-standardized DALYs rate in 195 countries and territories in 2017. Ethics approval and informed consent were not required for this study because of public accessibility to the data.

### Socioeconomic Status

The socio-demographic index (SDI) is a composite indicator of development status, containing total fertility rate, educational attainment, and lag-distributed income. The SDI varies from 0 to 1 strongly correlated with health outcomes, with a higher value indicating a higher level of socioeconomic development (22–24). Countries were categorized into five groups by their overall development status level according to the 2017 SDI values (25): high SDI (>0.81), high-middle SDI (0.70–0.81), middle SDI (0.61–0.70), low-middle SDI (0.46–0.60), and low SDI (<0.46).

### Health Inequalities

The Gini coefficient and the concentration index (CI) were used to quantify the magnitude of health inequalities in this study. Based on the Lorenz Curve, the Gini coefficient is a widely used measure of the extent of inequality (26, 27). The Gini coefficient ranges from 0 to 1. A region with perfect equality will have a value of 0, while a region with perfect inequality will have a value of 1 (28). The Gini coefficient is calculated by the age-standardized DALY rates owing to epilepsy from 195 countries to explore the trends in between-country health inequality from 1990 to 2017. The CI, derived from the concentration curve, is commonly used as an index to measure the socioeconomic-related inequalities (29). The CI is calculated by national age-standardized DALY rates and the corresponding SDI to measure the degree of health inequality associated with socioeconomic conditions. The CI ranges from −1 to 1. A value of 0 for CI means the absence of inequality related to socioeconomic characteristics. A positive (negative) value of the CI indicates that the epilepsy burden is more concentrated in countries with high (low) levels of socioeconomic development. The Gini coefficient was calculated by the INEQQERR module while the CI by the CONCINDC module by using STATA 15 (StataCorp, College Station, Texas, USA).

### Statistical Analyses

All statistics were presented as values with 95% uncertainty intervals (UIs). The Wilcoxon signed rank test is a non-parametric statistical hypothesis test when comparing two related samples, matched samples, or repeated measurements on a single sample to assess whether their population mean ranks differ. The Wilcoxon signed rank test should be used if the differences between pairs of data are non-normally distributed (30). The Pearson correlation coefficient measures the relative strength of the linear relationship between two variables. Pearson's *r* ranges from −1 to 1. An *r* of −1 indicates a perfect negative linear relationship, an *r* of 0 indicates no linear relationship, and an *r* of 1 indicates a perfect positive linear relationship between variables (31). The paired Wilcoxon signed rank test was utilized to compare gender differences in global age-standardized prevalence and DALY rates for each SDI-based country group. Association of gender difference (male minus female) and gender ratio (male to female) in age-standardized DALY rates with SDI was tested by Pearson correlation and linear regression analyses. All the analyses were conducted with IBM SPSS 23.0 Statistical software and Prism Software (version 8; GraphPad). A *p* < 0.05 was considered statistically significant.

## Results

### Global Trends and Gender Disparity of Epilepsy Burden

**Figure 1:**
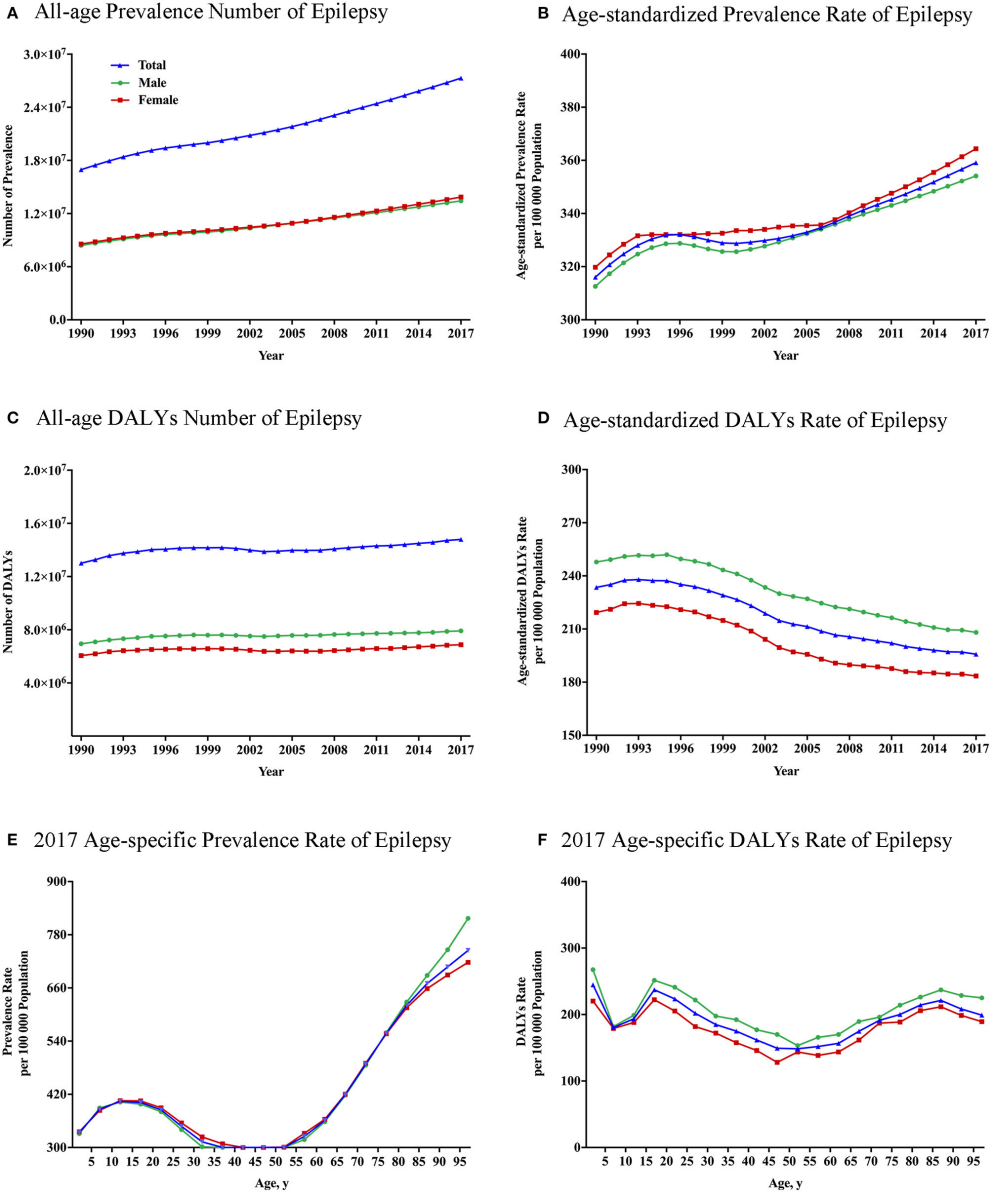
Global total and gender-specific burden of epilepsy by year and age. **(A)** All-age prevalence numbers from 1990 to 2017. **(B)** Age-standardized prevalence rates per 100,000 population from 1990 to 2017. **(C)** All-age DALY numbers from 1990 to 2017. **(D)** Age-standardized DALY rates per 100,000 population from 1990 to 2017. **(E)** Age-specific prevalence rate in 2017. **(F)** Age-specific DALY rate in 2017. DALYs, disability-adjusted life-years.

**Figure 2:**
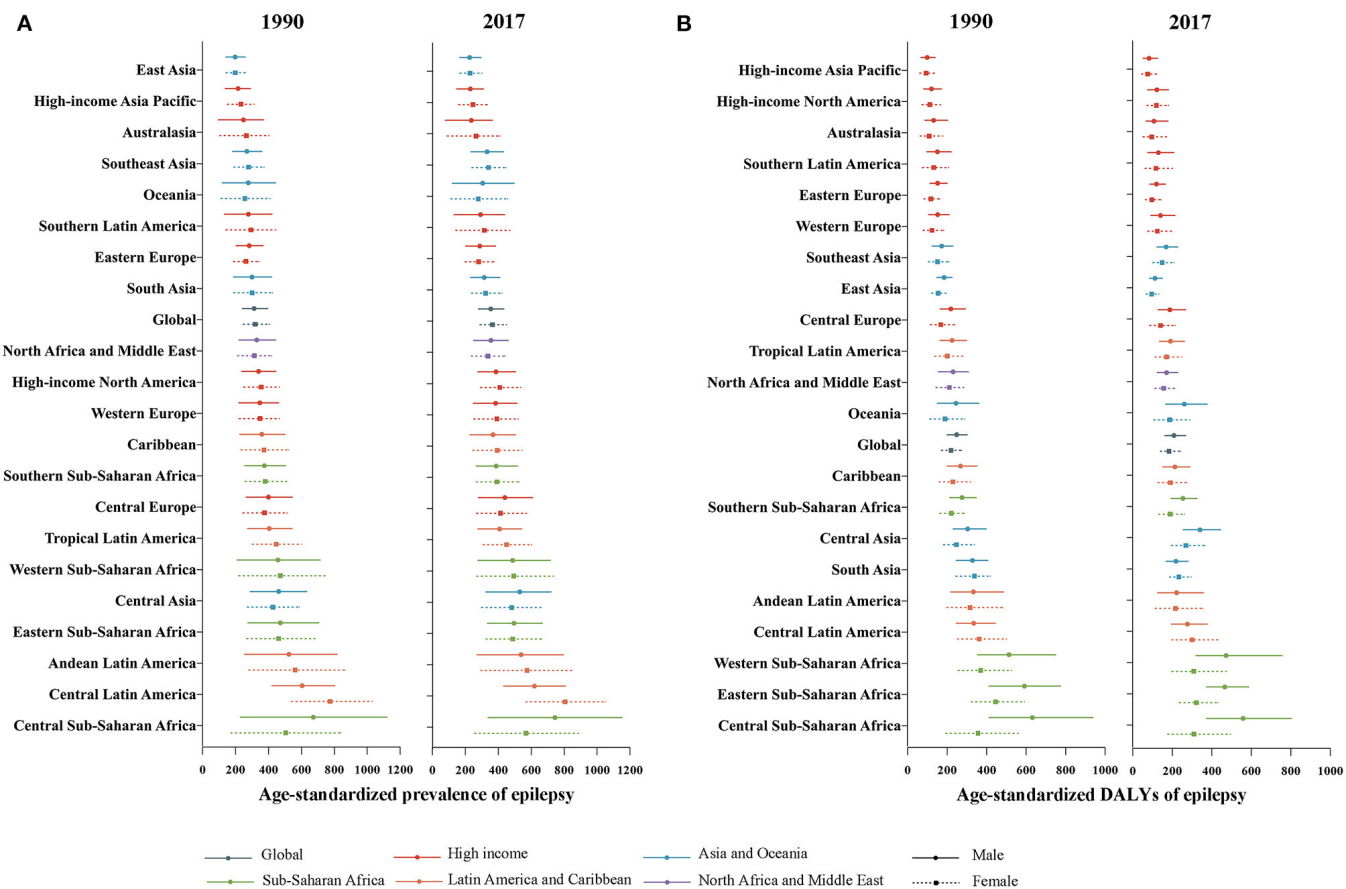
Global gender-specific burden of epilepsy by 21 GBD regions in 1990 and 2017. **(A)** Age-standardized prevalence rate. **(B)** Age-standardized DALYs rate. The *Y*-axis order is arranged according to the level of the age-standardized prevalence and DALYs rate of males. DALYs, disability-adjusted life-years.

The all-age prevalence number due to epilepsy increased by 60.6%, from 17.0 (95% UI: 13.0–21.5) million in 1990 to 27.3 (95% UI: 21.6–33.4) million in 2017 (Figure 1A). After controlling for the effect of population and age structure, age-standardized prevalence rate of epilepsy rose by 13.6%, from 316.0 (95% UI: 244.4–399.3) per 100,000 population in 1990 to 359.1 (95% UI: 283.8–441.4) per 100,000 population in 2017 (Figure 1B). Similarly, the all-age DALYs number owing to epilepsy increased from 13.0 (95% UI: 10.3–15.9) million in 1990 to 14.8 (95% UI: 11.4–19.0) million in 2017, with a rise of 13.8% (Figure 1C). After controlling for population and age structure, a similar decline was observed in the age-standardized DALY rate between 1990 and 2017 (Figure 1D). Age-standardized DALY rate fell by 16.1% from 233.5 (95% UI: 186.4–285.9) per 100,000 population in 1990 to 195.8 (95% UI: 151.4–251.8) per 100,000 population in 2017. Among 21 GBD regions, despite the substantial decline in DALY rates (Figure 2B; Supplementary Table 1), a slight rise in the age-standardized prevalence rate of epilepsy was observed for both sexes from 1990 to 2017 (Figure 2A).

As shown in Figure 1, gender disparities existed in global burden of epilepsy since 1990, in the aspect of absolute number (*p* < 0.001), crude rates (*p* < 0.001), and age-standardized rates (*p* < 0.001) of DALYs. The total DALY numbers were 7.9 (95% UI: 6.2–10.1) million in men and 6.8 (95% UI: 5.2–9.0) million in women in 2017. Similarly, male subjects had a higher epilepsy burden in the crude DALY rates (206.4 DALYs per 100,000 men vs. 180.7 DALYs per 100,000 women) and age-standardized DALY rates (208.1 DALYs per 100,000 men vs. 183.5 DALYs per 100,000 women) than females in 2017. In Figures 2A,B, the greatest gender gap in DALY rate was found in Central Sub-Saharan Africa (male 631.7 vs. female 356.3 in 1990, male 558.3 vs. female 309.7 in 2017), followed by Eastern Sub-Saharan Africa and Western Sub-Saharan Africa regions.

### Global Gender-Specific Epilepsy Burden by Age

Age-specific prevalence rates of epilepsy reached a peak in the 95+ age group [817.2 (95% UI: 717.7–744.6] (Figure 1E). Three peaks [1–4: 244.7 (95% UI: 195.2–313.0), 15–19: 237.3 (95% UI: 178.0–309.3), and 85–89 years: 221.2 (95% UI: 159.6–304.5)] and a trough [50–54 years: 148.5 (95% UI: 116.3–191.0)] were observed on the age-specific DALY rate in 2017 (Figure 1F). Higher age-specific DALY rates were observed in males at all age groups (*p* < 0.01).

### Geographic Variation and Socioeconomic Disparity in Epilepsy Burden

**Figure 3:**
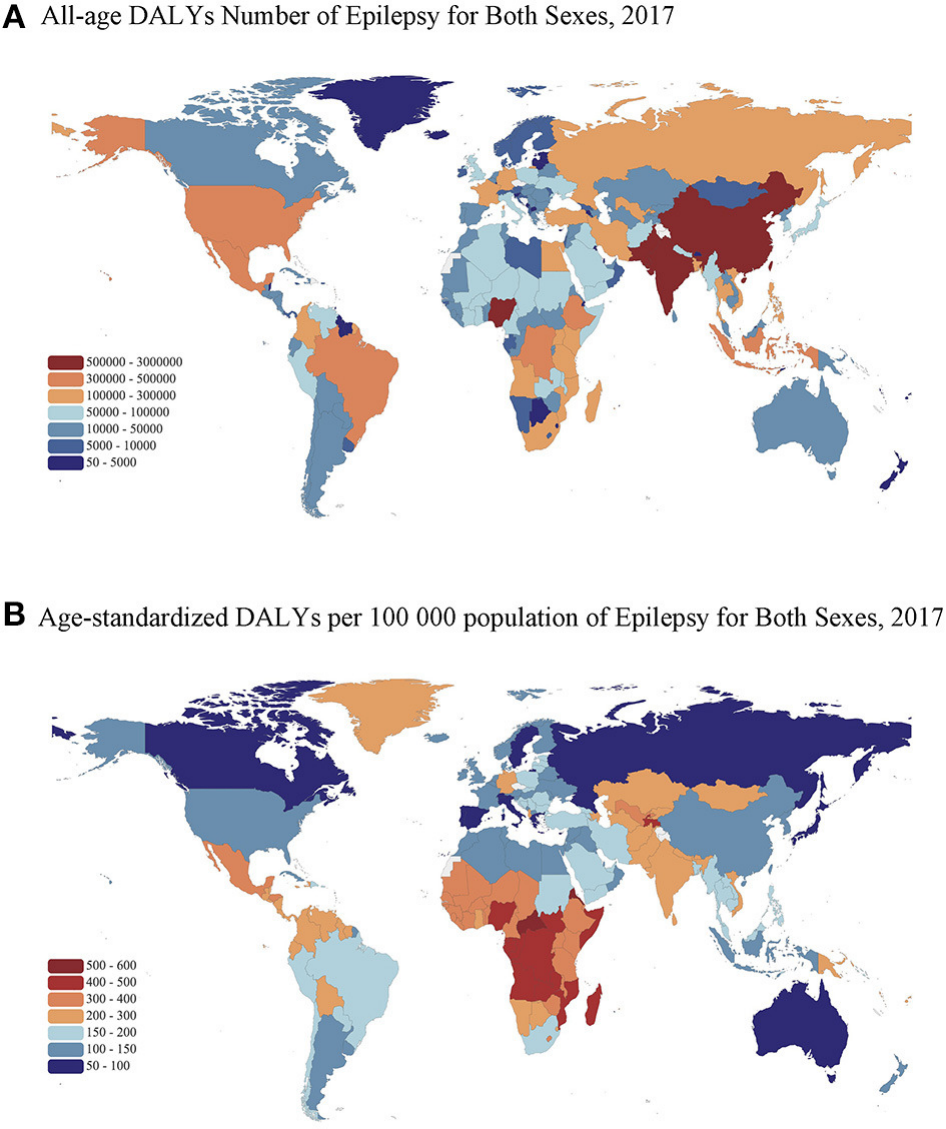
Maps of all-age DALY number and age-standardized DALYs rate of epilepsy in 2017 for both sexes. **(A)** All-age DALY number. **(B)** Age-standardized DALY rate per 100,000 population. DALYs, disability-adjusted life-years. The boundaries shown and the designations used on the maps do not imply the expression of any opinion of the authors.

Among the 195 countries and territories analyzed in the GBD 2017 study, there was considerable geographic variation in the epilepsy burden worldwide (Figure 3). The epilepsy burden concentrated in many Asian and African countries with a large population and low socioeconomic status. As shown in Figure 3A, DALYs number was greatest in India [2,984,664.7 (95% UI: 2,409,842.9–3,681,587.1)], followed by China, Nigeria, Pakistan, and United Status. Notably, the highest rate of age-standardized DALYs was concentrated in the Central African Republic [525.1 (95% UI: 291.2–832.7) per 100,000 population], followed by several African countries such as Eritrea, Angola, Mozambique, and Congo (Figure 3B). The lowest age-standardized DALY rate was concentrated in developed countries in Asia and Europe, such as Japan [68.0 (95% UI: 42.2–107.0) per 100,000 population], Singapore, Spain, Switzerland, and Sweden.

**Figure 4:**
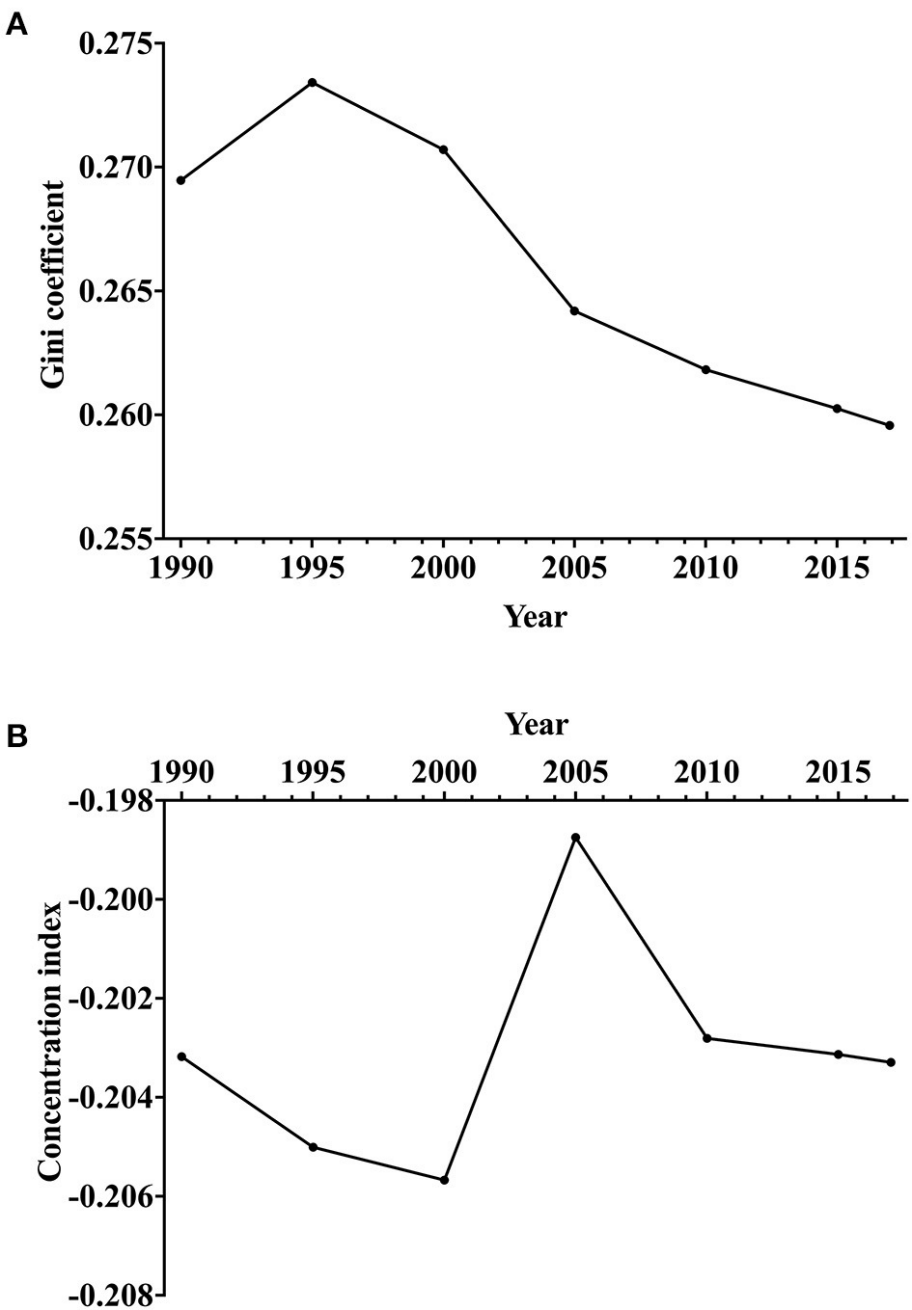
Trends of socioeconomic disparity in epilepsy burden in terms of age-standardized DALY rate across countries from 1990 to 2017. **(A)** Gini coefficient. **(B)** Concentration index. DALYs, disability-adjusted life-years.

Figure 4 shows the time trends of the Gini coefficient and CI. The Gini coefficients of epilepsy across countries decreased from 0.273 in 1995 to 0.259 in 2017 for the age-standardized DALY rate (Figure 4A), indicating that the between-country disparity of the epilepsy burden was declining. The negative values of the CI indicated that epilepsy burden was more concentrated in countries with lower socioeconomic development (Figure 4B).

**Figure 5:**
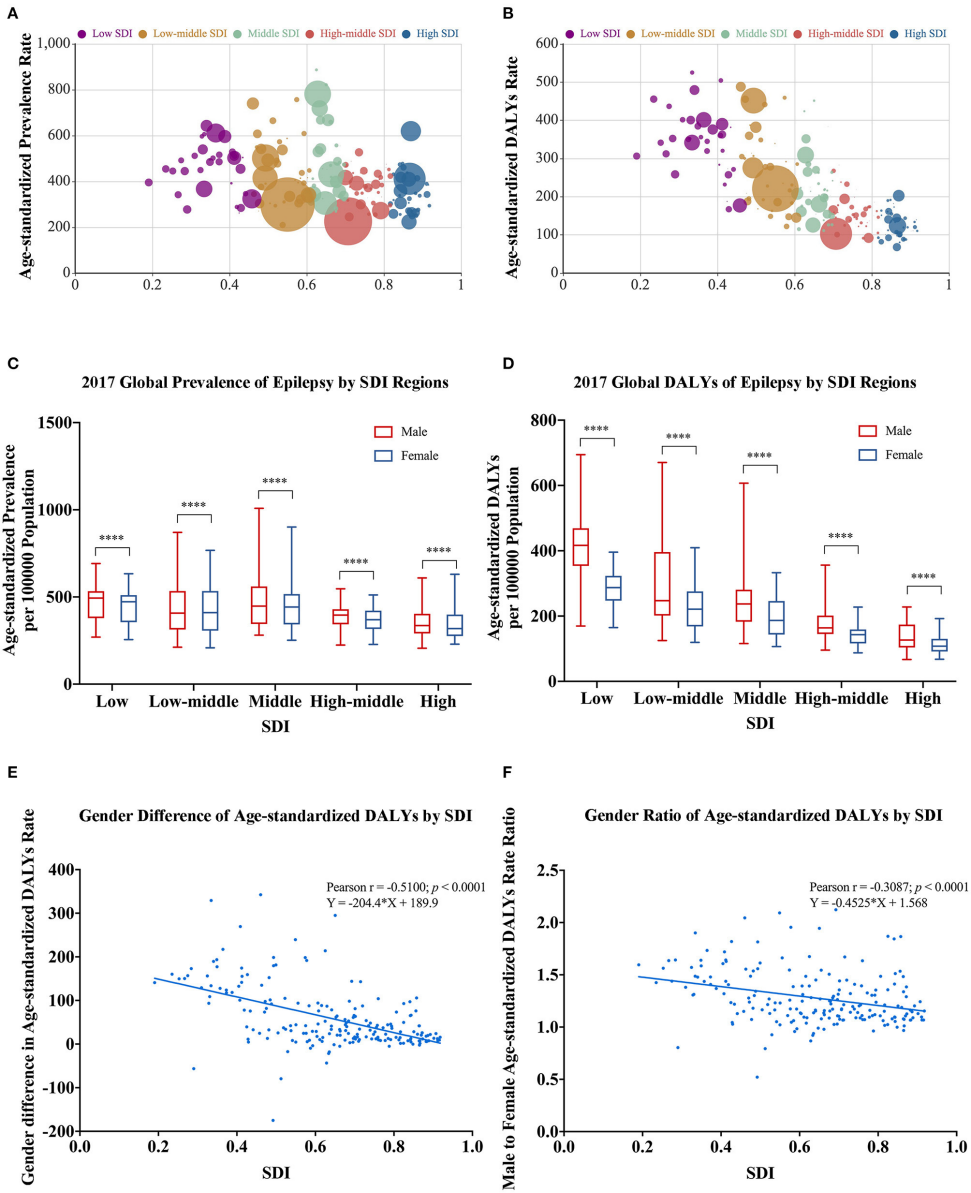
Gender-specific burden of epilepsy in different SDI regions in 2017. **(A)** All-age prevalence number and age-standardized prevalence rate (the area of the bubble represents the prevalence number). **(B)** All-age DALY number and age-standardized DALY rate (the area of the bubble represents the DALYs number). **(C)** Age-standardized prevalence rate by sex in different SDI groups. **(D)** Age-standardized DALY rate by sex in different SDI groups. **(E)** Association of gender difference (male minus female) in age-standardized DALY rates with SDI. **(F)** Association of gender ratio (male to female) in age-standardized DALY rates with SDI. DALYs, disability-adjusted life-years; SDI, socio-demographic index. ^*^For difference between sexes; ^****^p < 0.0001, paired Wilcoxon signed rank test.

The socio-demographic index (SDI) data in 2017 were available for 195 countries and territories, including 35 in the low SDI group, 41 in the low-middle SDI group, 40 in the middle SDI group, 41 in the high-middle SDI group, and 38 in the high SDI group. In Figures 5A,B, each bubble represents a country or a territory. The area of the bubble indicates the absolute burden number in the different SDI regions. The *Y* coordinate represents the age-standardized burden rate. The middle SDI and low-middle SDI regions suffered a higher burden in terms of prevalence numbers and age-standardized prevalence rates (Figure 5A), whereas the greatest DALY numbers and age-standardized DALY rates were located in the low SDI and low-middle regions (Figure 5B).

Males had higher age-standardized prevalence (*p* < 0.0001) and DALY rates (*p* < 0.0001) than females in all five SDI regions in 2017 (Figures 5C,D). Pearson correlation (*r* = −0.5100, *p* < 0.001) and linear regression analysis (*Y* = −204.4 × *X* + 189.9) indicated that gender differences (male minus female) in age-standardized DALY rates and SDI had a negative correlation (Figure 5E). Similarly, gender ratios (male to female) in age-standardized DALY rate were negatively associated with SDI in Pearson correlation (*r* = −0.3087, *p* < 0.001) and linear regression analysis (*Y* = −0.4525 × *X* + 1.568) (Figure 5F). Both sets of analyses showed that gender differences in epilepsy DALY rates were greater in countries with lower SDI.

## Discussion

Epilepsy has been a common public concern with a worldwide distribution. Our analysis of the GBD 2017 study presented that the global burden of epilepsy declined in terms of age-standardized DALYs over the past decades. Epilepsy caused a higher burden on adolescents and old people, especially in males. Countries with lower socioeconomic status and underdeveloped regions tended to have higher epilepsy burden and greater gender gap.

Epilepsy accounts for a significant proportion of the world's disease burden, with an increasing incidence in LMICs (49–215 per 1,000,000 people per year) (32–35). In this study, epilepsy caused 14.8 million DALYs, which accounted for 0.59% of total global DALYs in the GBD 2017 study. The DALYs numbers of epilepsy were greatest in India and China. The increasing burden in our results, as measured by the absolute number of DALYs, might be partly due to rapidly aging and growing population, rising life expectancy, and more risk factors (e.g., infections, birth injury, trauma, and stroke) (19, 23). A high burden of epilepsy has also been demonstrated in previous studies of specific regions [South Africa (7), China (8), and southeast Nigeria (36)] by using the DALY metrics. The number of patients with epilepsy is expected to rise further, as more than 5 million new cases are diagnosed each year (32). These findings necessitate more health-care approaches of government for the management of epilepsy.

Despite a steadily growing tendency in the DALY number of epilepsies, a reduction was observed in crude DALY rate and age-standardized DALY rate between 1990 and 2017. This is supported by a systematic analysis for the GBD 2016 study (37), which found a significant reduction in the mortality and DALY rates in patients with epilepsy globally from 1990 to 2016. Similarly, the age-standardized DALYs of all neurological disorders had an overall decrease between 1990 and 2015 (9). Factors contributing to the health improvement include continuing progress in treatment conditions, advanced medical technology, public awareness of epilepsy, and measures on epilepsy prevention by the government (6, 37).

As shown in Figure 1F, after controlling for the effect of population, the DALY rate of epilepsy in 2017 had three peaks in 1–4, 15–19, and 85–89 years old in 2017. These findings are partially consistent with those of several previous studies (9, 38). Epilepsy has a bimodal distribution according to age with peaks in the youngest individuals and the elderly (9, 23). The higher incidence of epilepsy in Europe occurs at 0–1 years old (39). The incidence of epilepsy in resource-rich countries is highest in the first few months of life, particularly in the immediate postnatal period, which falls significantly after the 1st year of life (40). The peak of DALY rate in young infants was probably due to perinatal hypoxia and trauma, metabolic disturbances, congenital malformations of the brain, and infection (19, 41). Inadequate perinatal care and high premature mortality are possible reasons for higher burden of epilepsy in infants. Perinatal and post-infective encephalopathy, cortical dysplasia, and hippocampal sclerosis account for the most severe symptomatic epilepsies in children (42). Sillanpää et al. (43) found a 21.7-fold risk of occurrence of a disability in children with epilepsy compared with controls.

It is worth noting that the DALY rate also reaches a peak in the adolescents in our findings. Similarly, the prevalence rate of epilepsy had a peak during adolescence in Figure 1E. A GBD study of epilepsy indicated that the years of life lost (YLLs) of idiopathic epilepsy peaked at age under 5 years and at age of 15–19 years (37). The probable etiology or risk factor for epilepsy depends on the age of the patient and the type of epilepsy (44). Myoclonic and absence seizures often occur between 5 and 15 years old. Two thirds of the cases with epilepsy were classified as idiopathic or cryptogenic, which is usually seen in the adolescents (45). In adolescents, idiopathic epilepsies account for the majority of cases, although trauma and infection play a role. Most idiopathic epilepsy syndromes have complex inheritance, probably because of interacting genetic and environmental factors (46). Furthermore, Infants and adolescents with epilepsy have difficulty in access to diagnosis and health care. Stigmatization and poor acceptability of epilepsy impact on the quality of life and long-term outcomes of infants and adolescents with epilepsy (47).

Moreover, the most common causes in the elderly are idiopathic epilepsy, trauma, head injury, alcohol abuse, brain tumors, and cerebrovascular disease (46). Epilepsy is associated with a number of age-related and aging-related diseases, such as Alzheimer's disease, dementias, stroke, and vascular and metabolic disorders (23).

Our study revealed that gender disparities in epilepsy have existed at a global level since 1990. In our study, significantly higher prevalence rates (*p* < 0.0001) and DALY rates (*p* < 0.0001) were found among males in different SDI regions. Pearson correlation and linear regression analysis indicated that the greater sexual differences in epilepsy DALY rates appeared in countries with lower SDI. Our findings provide data support for the gender research of epilepsy. Several studies indicated that the incidence of epilepsy was slightly higher in men than in women (11, 48–50). Steroid hormones might be the potential mechanism of gender differences in epilepsy (49). However, we have not found the specific biological basis of sex disparity in epilepsy in the previous literature (1, 11, 49), which needs further studying. It has been suggested that males are more susceptible to injury-induced seizures than females (49). Males have a higher lifetime risk of suffering from epilepsy, and this might be owing to men's occupation and their exposure to risk factors, such as head trauma and alcohol use. Additionally, because of the stigma and low family economic status in rural areas, females tend to have a lower consultation rate (10). Another survey (32) suggested that women were more likely to conceal the symptoms of epilepsy for sociocultural reasons in India.

More importantly, our findings pointed out that the burden of epilepsy is higher in many developing regions with low SDI and low-middle SDI, such as Sub-Saharan Africa, Latin America, and Central and South Asia. The negative CI indicated that countries with low levels of socioeconomic development had a higher epilepsy burden. Epilepsy has a significant economic impact on health-care needs, premature death, and disruption of work or education for individuals and their families (4, 51). A review of studies estimating the cost of epilepsy reported that the direct and indirect cost per person per year ranged from US$ 1,736 to 5,848 and 2,037 to 8,587, respectively (4). Several systematic studies (32, 37, 52) have reported that there was an interconnection between epilepsy and poverty. The lower socioeconomic status was related to poor health services, poor awareness of medical care, and infrequent outpatient clinic visits. The association between lower socioeconomic status with a higher burden of epilepsy in our study is in accordance with a review on epilepsy in Asia (6). The median numbers of neurologists in Asia, a region with great differences in economic development, were 0.03 and 2.96 per 100,000 population in low-income countries and high-income countries, respectively. The high treatment gap, which can be caused by inadequate health care response to epilepsy and unaffordable drug treatment, resulted in difficulties in epilepsy management in low-income regions. Moreover, the research showed that the epilepsy burden of epilepsy-related premature mortality is higher in LMICs than a burden in HICs (53). Lack of access to medical facilities and preventable risk factors (drowning, head injuries, and burns) in low-income countries have promoted the occurrence of epilepsy.

Poor knowledge of epilepsy and less education also affect the burden of epilepsy. People in rural areas of Asia and Africa tended to have more negative attitudes toward epilepsy than those in developed countries (54, 55). Besides, patients with active epilepsy living in low-income and middle-income countries receive no timely and effective treatment because the stigma and discrimination are difficult to overcome (56). The stigma can impact the quality of life and work for patients and their families. The burden in countries with low socioeconomic status can be reduced by further education about epilepsy and improved treatments, including antiepileptic drugs (AEDs).

Encouragingly, the analysis of the Gini coefficient indicated that the health disparity was shrinking worldwide. Comprehensive epilepsy care programs played an important role in this decrease. Relevant projects in many countries, such as China (57), India (58), and Brazil (59) have been carried out to reduce the burden of epilepsy and improve access to health-care services (11). Furthermore, the steady rise of CI between 2000 and 2005 may also be associated with the progress in health from the World Health Organization (WHO) Program. Since 1997, the Global Campaign Against Epilepsy was formed by the WHO, the International League against Epilepsy (ILAE), and the International Bureau for Epilepsy (IBE) (17). Between 2000 and 2003, WHO regional declarations on epilepsy were adopted to encourage country cooperation on reducing the epilepsy treatment gap.

To our knowledge, this study has provided a comprehensive assessment of the global burden of epilepsy at a regional level by using systematic and reliable GBD measures. Compared with the previous studies on the global burden of epilepsy, our study highlights and analyzes the gender and socioeconomic disparities of global burden of epilepsy concretely. We discussed not only the gender disparity by year, age, and region, but also the relationship between gender difference and ratio in the global burden of epilepsy with SDI. This is a more specific perspective of discovering the gender disparity of global burden of epilepsy. This study could help the decision-makers to pay more attention to gender and socioeconomic-related disparities in epilepsy burden. The long-term patterns in gender and socioeconomic-related disparities in epilepsy burden could be analyzed by the annual updates of the GBD study.

However, our study has several limitations. First, our research is subject to the GBD 2017 study and therefore has the same GBD methodological disadvantages, which have been described previously (21, 23, 24, 33). Although the GBD 2017 study provided estimates through hierarchical models by collecting data from representative population-based studies, the quality and availability of data are still limited. Therefore, the data sources and statistical assumptions may lead to bias in the included literature. The selection and publication bias may have contributed to the rise of CI between 2000 and 2005. Second, most epidemiological research on epilepsy has been done in high-income regions. There is an urgent need for more research in low SDI regions to raise awareness of epilepsy. Third, the impact of risk factors and specific classification of epilepsy on the epilepsy burden could be explored in a further research.

## Conclusion

The global epilepsy burden in terms of DALY rates had substantial declines in the past decades. However, continuous growth in DALY numbers indicates high costs of health care in the future owing to the aging population. Males, especially those who live in less developed countries, suffer a heavier burden of epilepsy than females. A higher burden of epilepsy has been found in countries with lower SDI. These findings could draw the attention to the gender difference and geographical distribution in global epilepsy burden and help to formulate health policies, especially in developing countries, to reduce the gender gap and burden of epilepsy.

## Data Availability Statement

The original contributions presented in the study are included in the article/Supplementary Material, further inquiries can be directed to the corresponding author/s.

## Author Contributions

YH, YS, and QD: literature search and data collection. YH, YS, QD, YD, and CS: analysis and interpretation. YH, YS, QD, YD, CS, SW, MD, and YX: drafting of manuscript. All authors: approved the final version.

## Conflict of Interest

The authors declare that the research was conducted in the absence of any commercial or financial relationships that could be construed as a potential conflict of interest.
