# Supplementary material for: Gender and Socioeconomic Disparities in Global Burden of Epilepsy: An Analysis of Time Trends From 1990 to 2017
Source: Front Neurol. 2021 Apr 16;12:643450. doi: 10.3389/fneur.2021.643450 (PMC8085398; doi:10.3389/fneur.2021.643450)
Supplement: Supplementary file 1 [file Table_1.DOC]

**Gender and** **Socioeconomic** **Disparities in Global Burden of Epilepsy: An Analysis of Time Trends from 1990 to 2017**

Yin Hu^1*^, Yi Shan^2*^, Qiang Du^1*^, Yao Ding^1^, Chunhong Shen^1^, Shuang Wang^1^, Meiping Ding^1#^, Yufeng Xu^2#^

^1^ Department of Neurology, the Second Affiliated Hospital, College of Medicine, Zhejiang University, 88 Jiefang Road, Hangzhou 310009, China

^2^ Department of Ophthalmology, the Second Affiliated Hospital, College of Medicine, Zhejiang University, 88 Jiefang Road, Hangzhou 310009, China

^*^ These authors have contributed equally to this work.

^#^ Co-corresponding authors

Corresponding Author:

Meiping Ding, MD, Department of Neurology, the Second Affiliated Hospital, College of Medicine, Zhejiang University, 88 Jiefang Road, Hangzhou, Zhejiang, 310009, China. E-mail address: [meipingd@163.com](mailto:meipingd@163.com). Tel.: +86 571 87784750.

Yufeng Xu, MD, PhD, Department of Ophthalmology, the Second Affiliated Hospital, College of Medicine, Zhejiang University, 88 Jiefang Road, Hangzhou, Zhejiang, 310009, China. Email: xuyufeng0216@zju.edu.cn. Tel.: +86-0571-87783897; Fax: +86-0571-87783897.

Correspondence and requests for data should be addressed to Yufeng Xu ([xuyufeng0216@zju.edu.cn)](mailto:xuyufeng0216@zju.edu.cn)).

**Supplementary Table 1. Age-Standardized DALY Rates (per 100 000 population) of epilepsy by gender among GBD Regions in 1990, 2005, and 2017.**

| **GBD 2017 Super Region** |  | **1990** |  |  | **2005** |  |  | **2017** |  |  |
| --- | --- | --- | --- | --- | --- | --- | --- | --- | --- | --- |
| **GBD 2017 Region** | **Male** | **Female** | **Total** | **Male** | **Female** | **Total** | **Male** | **Female** | **Total** | **%△**  **(1990-2017)** |
| **Central / East Europe and Central Asia** | 200.6 | 155.6 | 177 | 221.3 | 161.4 | 190.1 | 196 | 152.1 | 173.3 | -2.1 |
| Europe, Central | 218.6 | 167.6 | 192.7 | 212.3 | 145.9 | 178.5 | 187.4 | 139.6 | 163.2 | -15.3 |
| Europe, Eastern | 151.5 | 117.4 | 133.2 | 148.1 | 105.6 | 125.6 | 119.3 | 96.2 | 106.9 | -19.7 |
| Asia, Central | 303.6 | 247.1 | 274.3 | 392.7 | 310 | 350.2 | 340 | 268.7 | 303.6 | 10.7 |
| **High Income** | 131.5 | 114.3 | 122.8 | 128.5 | 113.7 | 121 | 122.7 | 113.3 | 117.9 | -4 |
| Asia Pacific, high income | 98.6 | 93.2 | 95.9 | 93 | 83.1 | 88 | 82 | 74.9 | 78.4 | -18.2 |
| Europe, Western | 151.8 | 122.7 | 137.1 | 152.2 | 126.8 | 139.4 | 140.1 | 124.1 | 132 | -3.7 |
| Australasia | 131.4 | 109.1 | 120.2 | 121.7 | 99.6 | 110.4 | 106.8 | 95.3 | 100.9 | -16.1 |
| North America, high income | 120.4 | 112.4 | 116.3 | 117.7 | 114.2 | 115.9 | 121.4 | 119.5 | 120.5 | 3.6 |
| Latin America, Southern | 150.7 | 131.8 | 140.9 | 130.5 | 115.3 | 122.6 | 129.2 | 117 | 122.9 | -12.8 |
| **Latin America / Caribbean** | 284.6 | 281 | 282.7 | 237 | 229.4 | 233 | 233.9 | 233.9 | 233.8 | -17.3 |
| Latin America, Tropical | 224.8 | 201.1 | 212.8 | 186.3 | 161.5 | 173.6 | 190.4 | 170.4 | 180.1 | -15.4 |
| Latin America, Central | 334.1 | 362.1 | 348.4 | 280.8 | 296.3 | 288.7 | 276.2 | 300.2 | 288.4 | -17.2 |
| Latin America, Andean | 332.8 | 316 | 324.3 | 256.8 | 235.6 | 246.2 | 221.6 | 215.8 | 218.8 | -32.5 |
| Caribbean | 267.6 | 228.7 | 247.8 | 218 | 191.7 | 204.6 | 212.9 | 189.8 | 201.1 | -18.8 |
| **Southeast / East Asia, and Oceania** | 179.6 | 152.2 | 166.2 | 147.4 | 126.7 | 137.2 | 130 | 112.7 | 121.5 | -26.9 |
| Asia, Southeast | 172.2 | 151 | 161.5 | 169.2 | 148 | 158.5 | 168.4 | 148.3 | 158.3 | -2 |
| Asia, East | 184.2 | 154.3 | 169.7 | 139.3 | 118.7 | 129.3 | 112.2 | 95.1 | 103.9 | -38.8 |
| **GBD 2017 Super Region** |  | **1990** |  |  | **2005** |  |  | **2017** |  |  |
| **GBD 2017 Region** | **Male** | **Female** | **Total** | **Male** | **Female** | **Total** | **Male** | **Female** | **Total** | **%△**  **(1990-2017)** |
| Oceania | 244.8 | 188.8 | 217.6 | 259.8 | 190.6 | 225.9 | 260.5 | 186.1 | 223.9 | 2.9 |
| **North Africa / Middle East** | 230 | 210.9 | 220.6 | 192.4 | 179.2 | 185.9 | 170.7 | 156.2 | 163.6 | -25.8 |
| North Africa / Middle East | 230 | 210.9 | 220.6 | 192.4 | 179.2 | 185.9 | 170.7 | 156.2 | 163.6 | -25.8 |
| **South Asia** | 327.9 | 338.4 | 333.1 | 272.9 | 267.5 | 270.3 | 217.8 | 233.1 | 225.2 | -32.4 |
| Asia, South | 327.9 | 338.4 | 333.1 | 272.9 | 267.5 | 270.3 | 217.8 | 233.1 | 225.2 | -32.4 |
| **Sub-Saharan Africa** | 529.7 | 380.5 | 453.7 | 498 | 334.6 | 414.7 | 461.4 | 303.7 | 380 | -16.2 |
| Sub-Saharan Africa, Southern | 275.4 | 220.8 | 246.7 | 343.8 | 272 | 304.8 | 253.7 | 188.7 | 219.5 | -11 |
| Sub-Saharan Africa, Eastern | 591.4 | 445.4 | 517 | 524.9 | 355.7 | 437.9 | 465.2 | 321.6 | 390.4 | -24.5 |
| Sub-Saharan Africa, Central | 631.7 | 356.3 | 491.1 | 573.9 | 317 | 440.6 | 558.3 | 309.7 | 429 | -12.6 |
| Sub-Saharan Africa, Western | 512.8 | 369.7 | 442.7 | 487.5 | 335.3 | 412.4 | 472 | 308.9 | 388.6 | -12.2 |

GBD, global burden of disease. DALYs, disability-adjusted life years. %△: the percent change of DALYs rate from 1990 to 2017.

Red color indicates higher DALYs rates, green indicates lower values, respectively.
